# Supplementary figures and images for: Spatial proteomics of Onchocerca volvulus with pleomorphic neoplasms shows local and systemic dysregulation of protein expression
Source: PLoS Negl Trop Dis. 2025 Mar 31;19(3):e0012929. doi: 10.1371/journal.pntd.0012929 (PMC11981190; doi:10.1371/journal.pntd.0012929)

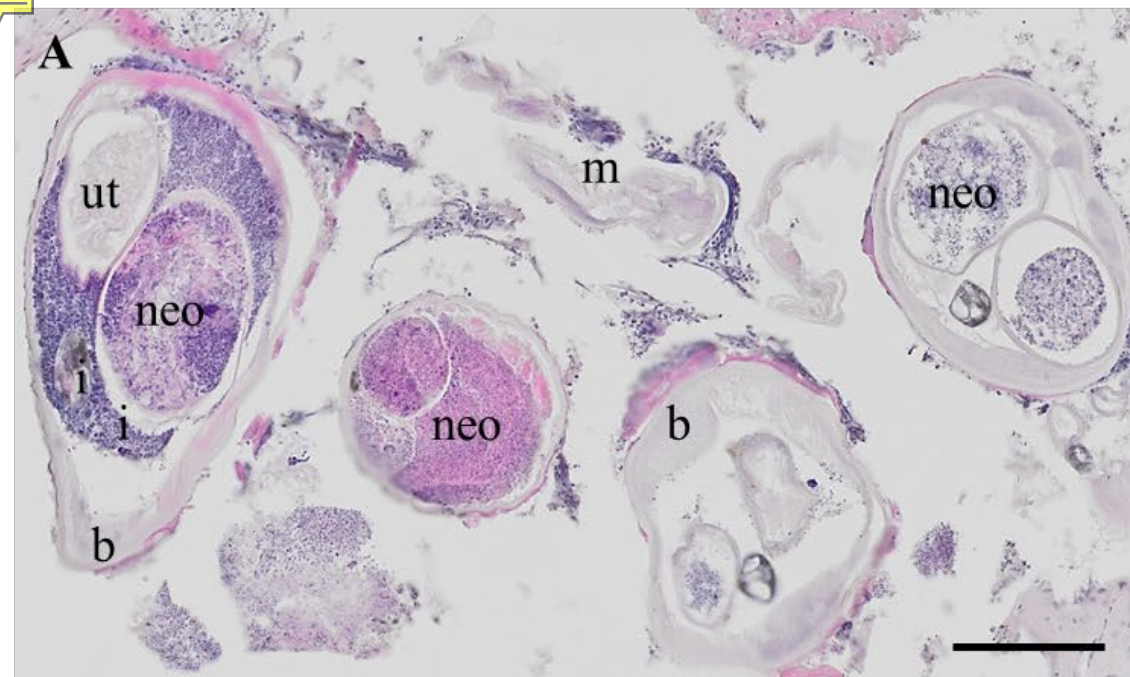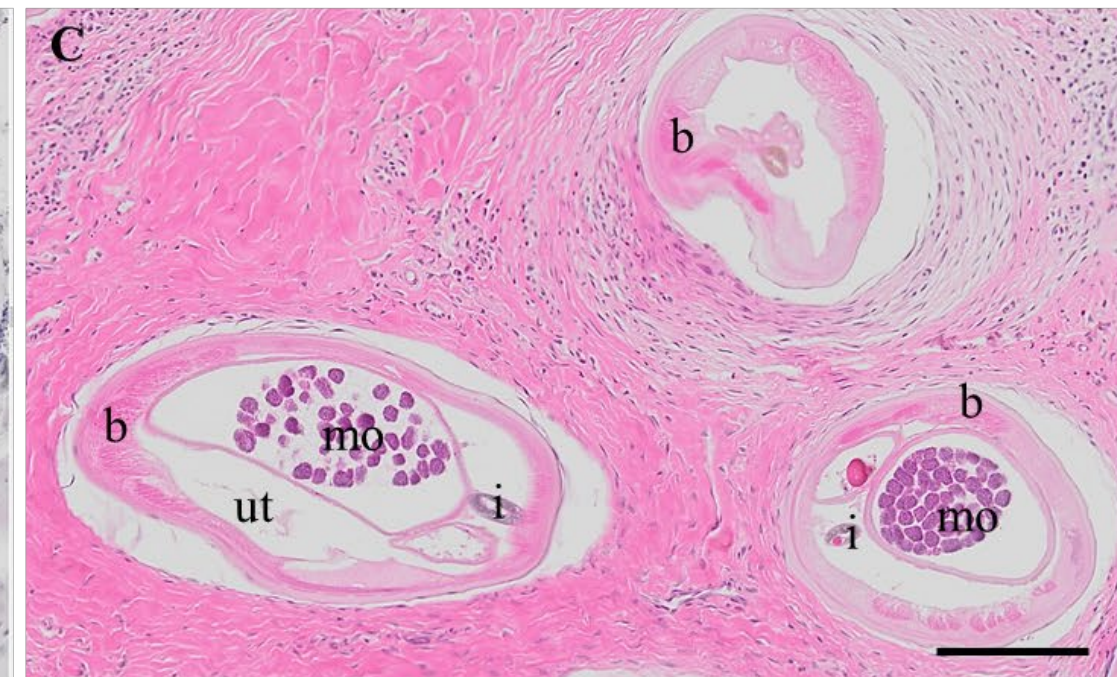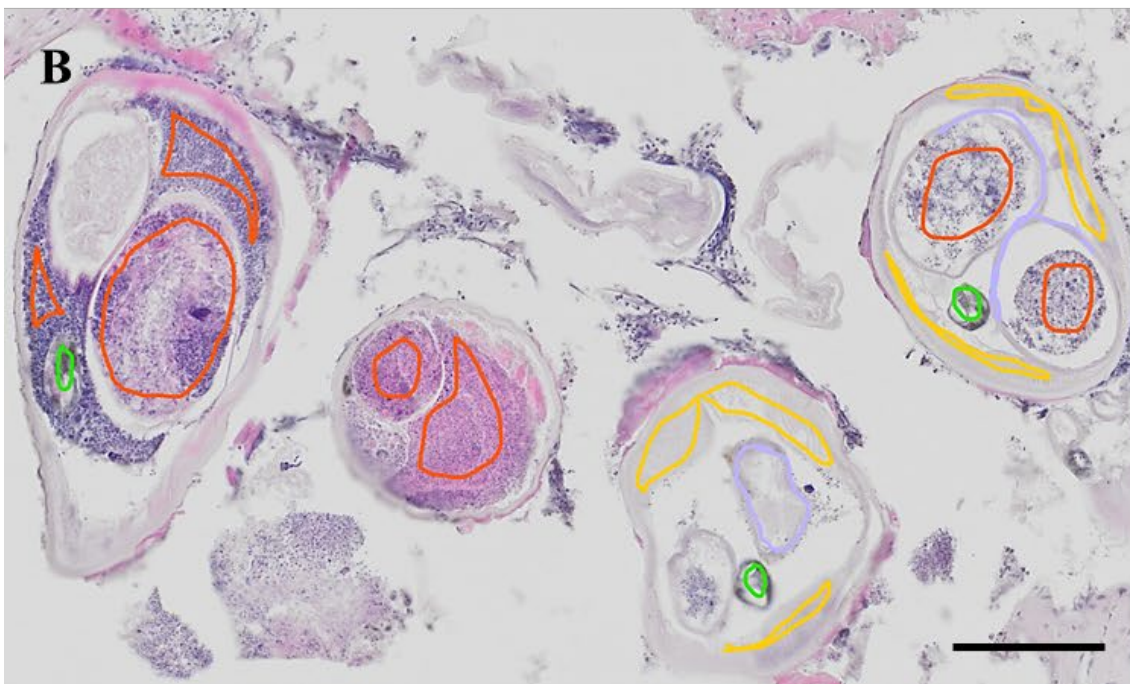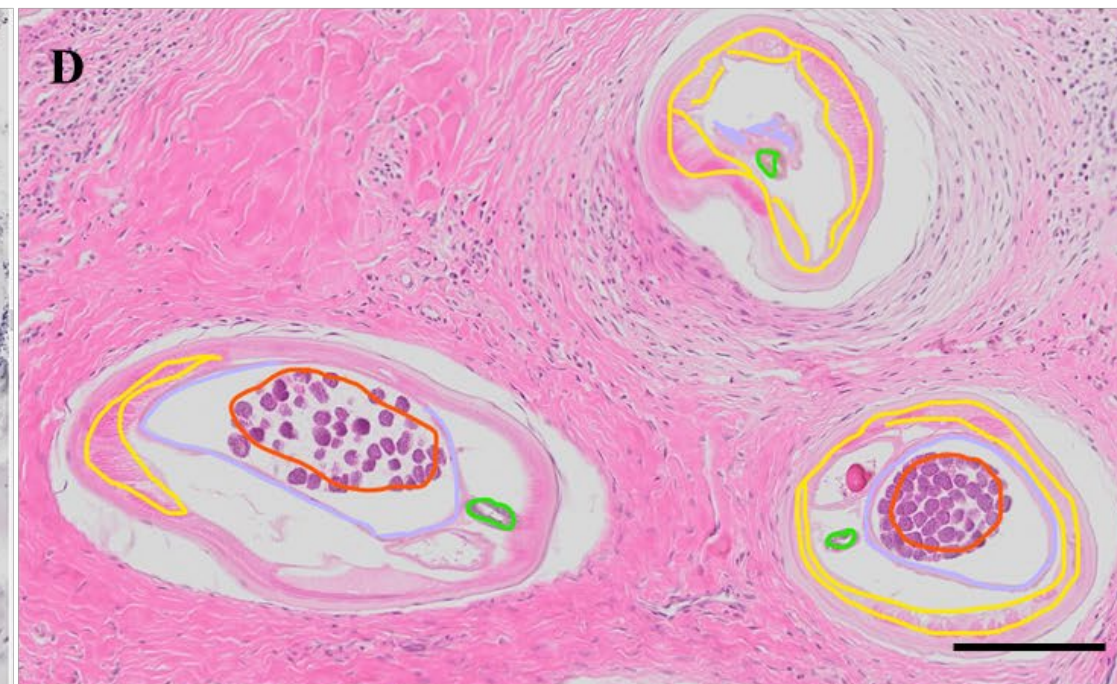

Supplement: S1 Fig — Worm sections which were used for LCM and which material was dissected using an image which had been manually color-coded to help keeping track which tissue should be dissected with the laser. A is a pleomorphic neoplasm worm and C is a healthy female. B and D are examples of images used at the LCM. Ut= uterus, b= bodywall, neo= neoplasm, m= male mo=morulae, Orange= neoplasm or embryos, green= gut; yellow= body wall, periwinkle= uterus wall. (PDF) [file pntd.0012929.s005.pdf]
