# Supplementary material for: Spatial proteomics of Onchocerca volvulus with pleomorphic neoplasms shows local and systemic dysregulation of protein expression
Source: PLoS Negl Trop Dis. 2025 Mar 31;19(3):e0012929. doi: 10.1371/journal.pntd.0012929 (PMC11981190; doi:10.1371/journal.pntd.0012929)
Supplement: S2 Fig — The sequence for OVOC8391 and its filarial orthologues were retrieved from WormBase Parasite. MegAlign Pro from DNA Star was used to align the sequences using OVOC8391 as reference. (PDF) [file pntd.0012929.s006.pdf]

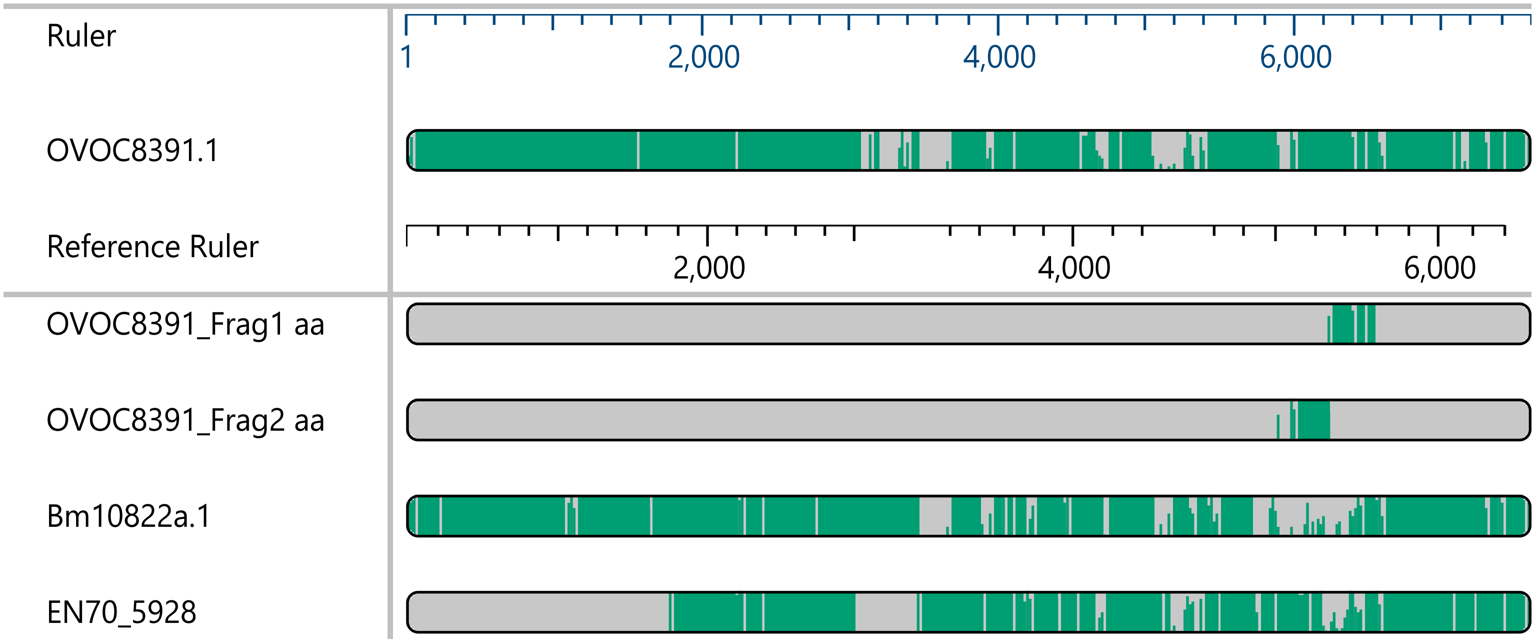

Ruler
1
2,000
4,000
6,000
OVOC8391.1
Reference Ruler
2,000
4,000
6,000
OVOC8391_Frag1 aa
OVOC8391_Frag2 aa
Bm10822a.1
EN70_5928

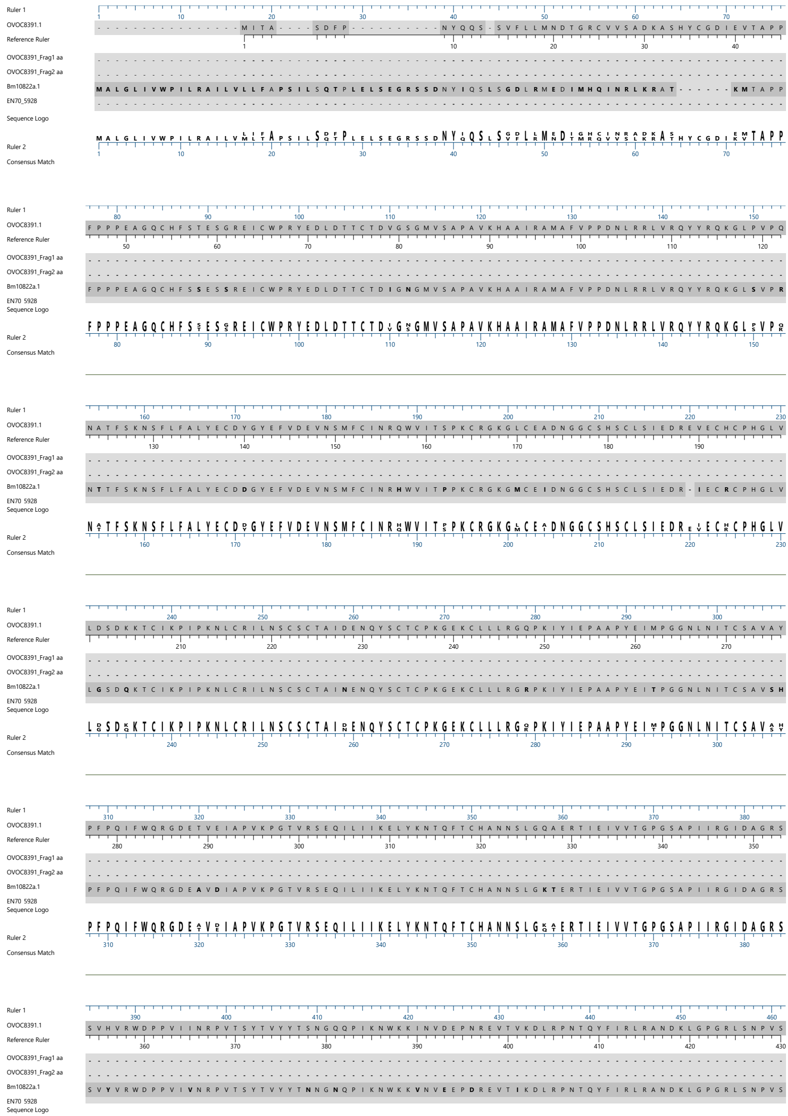

Ruler 1
OVOC8391.1
Reference Ruler
OVOC8391_Frag1 aa
OVOC8391_Frag2 aa
Bm10822a.1
EN70_5928
Sequence Logo
Ruler 2
Consensus Match
OVOC8391.1: ----------------MITA----SDFP----------NYQQS-SVFLLMNDTGRCVVSADKASHYCGDIEVTAPP
Bm10822a.1: MALGLIVWPILRAILVLLFAPSILSQTPLELSEGRSSDNYIQSLSGDLRMEDIMHQINRLKRAT------KMTAPP
OVOC8391.1: FPPPEAGQCHFSTESGREICWPRYEDLDTTCTDVGSGMVSAPAVKHAAIRAMAFVPPDNLRRLVRQYYRQKGLPVPQ
Bm10822a.1: FPPPEAGQCHFSSESSREICWPRYEDLDTTCTDIGNGMVSAPAVKHAAIRAMAFVPPDNLRRLVRQYYRQKGLSVPR
OVOC8391.1: NATFSKNSFLFALYECDYGYEFVDEVNSMFCINRQWVITSPKCRGKGLCEADNGGCSHSCLSIEDREVECHCPHGLV
Bm10822a.1: NTTFSKNSFLFALYECDDGYEFVDEVNSMFCINRHWVITPPKCRGKGMCEIDNGGCSHSCLSIEDR-IECRCPHGLV
OVOC8391.1: LDSDKKTCIKPIPKNLCRILNSCSCTAIDENQYSCTCPKGEKCLLLRGQPKIYIEPAAPYEIMPGGNLNITCSAVAY
Bm10822a.1: LGSDQKTCIKPIPKNLCRILNSCSCTAINENQYSCTCPKGEKCLLLRGRPKIYIEPAAPYEITPGGNLNITCSAVSH
OVOC8391.1: PFPQIFWQRGDETVEIAPVKPGTVRSEQILIIKELYKNTQFTCHANNSLGQAERTIEIVVTGPGSAPIIRGIDAGRS
Bm10822a.1: PFPQIFWQRGDEAVDIAPVKPGTVRSEQILIIKELYKNTQFTCHANNSLGKTERTIEIVVTGPGSAPIIRGIDAGRS
OVOC8391.1: SVHVRWDPPVIINRPVTSYTVYYTSNGQQPIKNWKKINVDEPNREVTVKDLRPNTQYFIRLRANDKLGPGRLSNPVS
Bm10822a.1: SVYVRWDPPVIVNRPVTSYTVYYTNNGNQPIKNWKKVNVEEPDREVTIKDLRPNTQYFIRLRANDKLGPGRLSNPVS

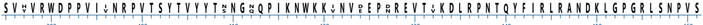

Ruler 2	390 400 410 420 430 440 450 460
Consensus Match	
Ruler 1	470 480 490 500 510 520 530
OVOC8391.1	V N T R K P A T R P Q L F I Q Q G D T I N V P P L K K F S I S C N V T R G D P S P K I A W Y T R A R P I N K P Q R K K F I T M E H G G L Y E S T N F S C V
Reference Ruler	440 450 460 470 480 490 500
OVOC8391_Frag1 aa	- -
OVOC8391_Frag2 aa	- -
Bm10822a.1	**L** N T R **R** P A **V** R P Q L F I Q **E** G D T I **Y** V **G** P L K **R** F S I S C N V T R G D P S P K I A W Y T R A R P I N K **S** Q R K K F I T M E H G G L Y E S T N F S C V
EN70 5928 Sequence Logo	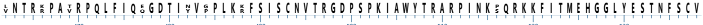
Ruler 2	470 480 490 500 510 520 530
Consensus Match	
Ruler 1	540 550 560 570 580 590 600 610
OVOC8391.1	A E N E A G R T T K R I Q V I V T G P T S P E R I R Y Q I H G N N V D L Q W E P P R I T N G Q L K D Y E V L Y T D N P S L P E E Y W E V A K A G S P D A R
Reference Ruler	510 520 530 540 550 560 570 580
OVOC8391_Frag1 aa	- -
OVOC8391_Frag2 aa	- -
Bm10822a.1	A E N **D** A G R T T K R I Q V I V T G P T S P E R I R Y Q I H G N N V D L Q W E P P R I T N G Q L K D Y **D** V L Y T D N P **K** L **S** **D** E Y W E V **V** K **T** G **N** P D A R
EN70 5928 Sequence Logo	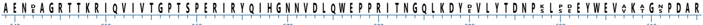
Ruler 2	540 550 560 570 580 590 600 610
Consensus Match	
Ruler 1	620 630 640 650 660 670 680 690
OVOC8391.1	S L Q I P N L K E K Q D Y T F K I R A Q S D L G P G L F S D Q F T L T T W L A A R P P V V T I T P A E K I V K D P S N E E L V F E C E A I G V P R P K I L
Reference Ruler	590 600 610 620 630 640 650 660
OVOC8391_Frag1 aa	- -
OVOC8391_Frag2 aa	- -
Bm10822a.1	S L **R** I **S** N L K E K **R** D Y T F K I R A **H** **N** **E** L G P G L F S **N** Q F T L T T W L A A R P P **I** V T I T P A E K **V** V K D P S N E E L **I** F E C E A I G V P R P K I L
EN70 5928 Sequence Logo	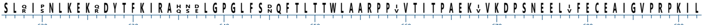
Ruler 2	620 630 640 650 660 670 680 690
Consensus Match	
Ruler 1	700 710 720 730 740 750 760
OVOC8391.1	W L W S G G L V E D G K N E F R I Y D V T P P D A Q D R S N S K L I A E K T N R A G V A T C Q A V N A H G S D E K R T E V K I V G P G S P P R D I S T V P
Reference Ruler	670 680 690 700 710 720 730
OVOC8391_Frag1 aa	- -
OVOC8391_Frag2 aa	- -
Bm10822a.1	W L W S G G L V E D G K N E F R I Y D V T P P D A Q **N** R S N S K L I A E K T N R A G V A T C Q A V N **P** H G S D E K **H** T E V K I V G P G S P P R D I S T **I** P
EN70 5928 Sequence Logo	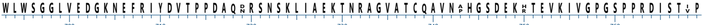
Ruler 2	700 710 720 730 740 750 760
Consensus Match	
Ruler 1	780 790 800 810 820 830 840
OVOC8391.1	F T N S F Q V S W N P P K Y P N G K I T R Y I I Y Y T K N P D D A L G D W E T T S V E G D Q L E V T I P A D E D T P Y N V R V Q A A T K D G L G I V S E V
Reference Ruler	740 750 760 770 780 790 800 810
OVOC8391_Frag1 aa	- -
OVOC8391_Frag2 aa	- -
Bm10822a.1	F T N **D** F **K** V S W N P P K Y P N G K I **I** R Y I I Y Y T K N P D D **L** L G D W E **K** T **A** V **G** G D Q L E V T **V** **S** A D E D T P Y N V R V Q A **V** T K D G **P** G I **I** S E **A**
EN70 5928 Sequence Logo	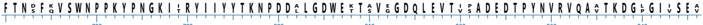
Ruler 2	780 790 800 810 820 830 840
Consensus Match	
Ruler 1	850 860 870 880 890 900 910 920
OVOC8391.1	Y D V T T G K K P I P L S V K L V V L D P Q V R E G D T E T I V E P R Q T I I F K C D A K G R P A P Q I S Y T W L P F N D T E S G Q E P T P I P A Y S D P
Reference Ruler	820 830 840 850 860 870 880 890

```
OVOC8391_Frag1 aa   ------------------------------------------------------------------------------
OVOC8391_Frag2 aa   ------------------------------------------------------------------------------
Bm10822a.1          YDVTTGKKPVPLSVKLVILDPQVEDGSTETVVEPLKTIIFKCDAKGRPAPQISYTWLPFNETESGQEPISIPAYSDP
EN70 5928 Sequence Logo
Ruler 2             850  860  870  880  890  900  910  920
Consensus Match
```

```
Ruler 1             930  940  950  960  970  980  990
OVOC8391.1          QEAHRYHSLDVNTNTATKRALMCSARNVDGSVSDRHIFNVIKPGSPPENVQAIIDPDNRVTISWQEPKYPNGPIIKY
Reference Ruler     900  910  920  930  940  950  960
OVOC8391_Frag1 aa   ------------------------------------------------------------------------------
OVOC8391_Frag2 aa   ------------------------------------------------------------------------------
Bm10822a.1          QEAHRYHSLDVNTNTATKRALMCNARNIDGSESDQHIFNVLKPGSPPENIQAIIDLDNRVTMSWQEPKYPNGPIVKY
EN70 5928 Sequence Logo
Ruler 2             930  940  950  960  970  980  990
Consensus Match
```

```
Ruler 1             1,010  1,020  1,030  1,040  1,050  1,060  1,070
OVOC8391.1          KVYVTSDPAEPISNWQIYDAEPSEAPNLILERGELQPETPYYVKIAAVGQGGEGLQSDVALFETVSGGILFLMDSFV
Reference Ruler     980  990  1,000  1,010  1,020  1,030  1,040
OVOC8391_Frag1 aa   ------------------------------------------------------------------------------
OVOC8391_Frag2 aa   ------------------------------------------------------------------------------
Bm10822a.1          KVYLTSDPSEPINHWNIYDVEPSEEPRLVLERGKLQPETPYYIKVVAVGEKGEGLQSDIILFETVSG----------
EN70 5928 Sequence Logo
Ruler 2             1,010  1,020  1,030  1,040  1,050  1,060  1,070
Consensus Match
```

```
Ruler 1             1,080  1,090  1,100  1,110  1,120  1,130  1,140  1,150
OVOC8391.1          LSNTVMSTTVSSALSQKQLRKLLKAPIDAPKDVLATVEQDNTMNISWTGPSVPNGPIQACLLESYTVYFTPLDQHQT
Reference Ruler     1,050  1,060  1,070  1,080  1,090  1,100  1,110  1,120
OVOC8391_Frag1 aa   ------------------------------------------------------------------------------
OVOC8391_Frag2 aa   ------------------------------------------------------------------------------
Bm10822a.1          ------------------------APIDEPKNVLATVERDNTMDISWTGPSVPNGPIQA-----YTVYFTPLDQYQT
EN70 5928 Sequence Logo
Ruler 2             1,080  1,090  1,100  1,110  1,120  1,130  1,140  1,150
Consensus Match
```

```
Ruler 1             1,160  1,170  1,180  1,190  1,200  1,210  1,220  1,230
OVOC8391.1          SDDAYKQWDKIVVQSNDDFGTLRLNKDIYDILPNRLYRIRVSATNDQSEGPASEPITVRTESGETPPIIRLEPGDNP
Reference Ruler     1,130  1,140  1,150  1,160  1,170  1,180  1,190
OVOC8391_Frag1 aa   ------------------------------------------------------------------------------
OVOC8391_Frag2 aa   ------------------------------------------------------------------------------
Bm10822a.1          SDDAYKQWDKIVVQSSENFGTLRLNKDIYDILPNKLYRIRVSATNDLSEGPASEPITIRTESGETPPVIHLEPIDNP
EN70 5928 Sequence Logo
Ruler 2             1,160  1,170  1,180  1,190  1,200  1,210  1,220  1,230
Consensus Match
```

```
Ruler 1             1,240  1,250  1,260  1,270  1,280  1,290  1,300
OVOC8391.1          ATVAPYGSISVRCTATGIPPPNIMWIIGTNASDVIRGPILQLTDLQKDETATCKAENNAGQTQEVLQILVAGPGSPP
Reference Ruler     1,210  1,220  1,230  1,240  1,250  1,260  1,270
OVOC8391_Frag1 aa   ------------------------------------------------------------------------------
OVOC8391_Frag2 aa   ------------------------------------------------------------------------------
Bm10822a.1          LTVAPYGSISVRCTATGIPPPIIMWIIGTNASDVIRGPILQLTNLRKDETVTCKAENNAGQAQEVLQILVAGPGTPP
EN70 5928 Sequence Logo
Ruler 2             1,240  1,250  1,260  1,270  1,280  1,290  1,300
Consensus Match
```

Ruler 1: 1,310 1,320 1,330 1,340 1,350 1,360 1,370 1,380

OVOC8391.1: N E I V A L P L D N Q Q V N I E W T T P D N P N G K I T D Y I I H Y G E I L D G E T I P P T W E T V R V S A D E P Q K Y R L T D L K P K T N Y A I R L Q A

Reference Ruler: 1,280 1,290 1,300 1,310 1,320 1,330 1,340 1,350

OVOC8391_Frag1 aa: -

OVOC8391_Frag2 aa: -

Bm10822a.1: N E I V A **F** P L **E** N Q Q **A S** I E W T T P D **S** P N G **R** I T D Y I I H Y G E **V F** D G E **A L** P **R A** W E T V **H** V S A **E** E P Q K **H** R L **V G** L K P K T **S** Y A I R L Q A

EN70 5928

Sequence Logo

Ruler 2: 1,310 1,320 1,330 1,340 1,350 1,360 1,370 1,380

Consensus Match

Ruler 1: 1,390 1,400 1,410 1,420 1,430 1,440 1,450 1,460

OVOC8391.1: I S D R G R G V L S D P I K V T T L P L A P A M V Q P A D V K V H D N N T V A I Q F Q A P R D P E D P E K P I K E F V V H Y T D D D P S S D D A E W K E M

Reference Ruler: 1,360 1,370 1,380 1,390 1,400 1,410 1,420 1,430

OVOC8391_Frag1 aa: -

OVOC8391_Frag2 aa: -

Bm10822a.1: **M** S D R G **P** G V L S D P I K **I** T T L P L A P A **I** V Q **L S** D **I** K V H **E** N N T V A I Q F **D** A P R D P E D P **G** K P I K E F **L I** H Y T D D D P **L** S **E** D A E W K E M

EN70 5928

Sequence Logo

Ruler 2: 1,390 1,400 1,410 1,420 1,430 1,440 1,450 1,460

Consensus Match

Ruler 1: 1,470 1,480 1,490 1,500 1,510 1,520 1,530

OVOC8391.1: S W T E P D D D F S V N I P I G G E H F K P D T K Y S I K I T A R G E I D S P P S E P V V F Q T G D G I V P P E K P Q I N V D V P D N V I R V P A G S D Y

Reference Ruler: 1,440 1,450 1,460 1,470 1,480 1,490 1,500

OVOC8391_Frag1 aa: -

OVOC8391_Frag2 aa: -

Bm10822a.1: **F** W T E P D D D F S V **S** I P I G G E H F K P D T K Y S I K **L I** A R G E I D S P P S E P **I** V F Q T G D G I V P P E K P Q I N V D **A** P D **D** V I R V P A G S D Y

EN70 5928

Sequence Logo

Ruler 2: 1,470 1,480 1,490 1,500 1,510 1,520 1,530

Consensus Match

Ruler 1: 1,550 1,560 1,570 1,580 1,590 1,600 1,610

OVOC8391.1: T V G C S S S G F P P P R I F W - - - - - - P L S D G P M L R L Q D V K E T V K A K C V A E N E G G R A E T D F E I F V T G P G N A P S N I R L N S L R P

Reference Ruler: 1,510 1,520 1,530 1,540 1,550 1,560 1,570

OVOC8391_Frag1 aa: -

OVOC8391_Frag2 aa: -

Bm10822a.1: T V G C S S **T** G F P **I** P R I F W **V N K D E Q** P L S D G P M L R L Q D V K E T V K A K C V A E N E G G R **T** E T **P** F E I F V T G P G N A P S N I R L N S **V** R P

EN70 5928

Sequence Logo

Ruler 2: 1,550 1,560 1,570 1,580 1,590 1,600 1,610

Consensus Match

Ruler 1: 1,620 1,630 1,640 1,650 1,660 1,670 1,680 1,690

OVOC8391.1: R T I H I D W D P P T I P N G N I T R Y I I Y Y T P L D D Q A S L F C K A L I Y Q M G Q I P K K P I T E W M T F H K D S E P L M G L P Q R A D L V D F I E

Reference Ruler: 1,590 1,600 1,610 1,620 1,630 1,640 1,650

OVOC8391_Frag1 aa: -

OVOC8391_Frag2 aa: -

Bm10822a.1: R T I H I D W D P P T I P N G N I T R Y I I Y Y T P L D D Q - - - - - - **N I V** Y Q M G Q I P K K P I T E W M T **S** H K D S E P L **I** G **A S** Q R A D L **I** D F **V** E

EN70 5928

Sequence Logo

Ruler 2: 1,620 1,630 1,640 1,650 1,660 1,670 1,680 1,690

Consensus Match

Ruler 1: 1,700 1,710 1,720 1,730 1,740 1,750 1,760

OVOC8391.1: P D T A Y A V V L Q A V N Q D G P G P Y S E Q H T I R T M S R A R E G P P L D L K V E P E G Q R S A N V E W Q K P V T S E Q P P I G Y E L Y Y I K A D A K

Reference Ruler: 1,660 1,670 1,680 1,690 1,700 1,710 1,720 1,730

OVOC8391_Frag1 aa: -

OVOC8391_Frag2 aa: -

Bm10822a.1: P D T A Y A V V L Q A V N Q D G P G P Y S E Q H T I R T M S R A R E G P P L D L K V E P E G Q R S A N V E W **H R** P **I** T S E Q **L** P I G Y E L Y Y I K A D A K

EN70 5928

Sequence Logo

Ruler 2: 1,700 1,710 1,720 1,730 1,740 1,750 1,760

Consensus Match

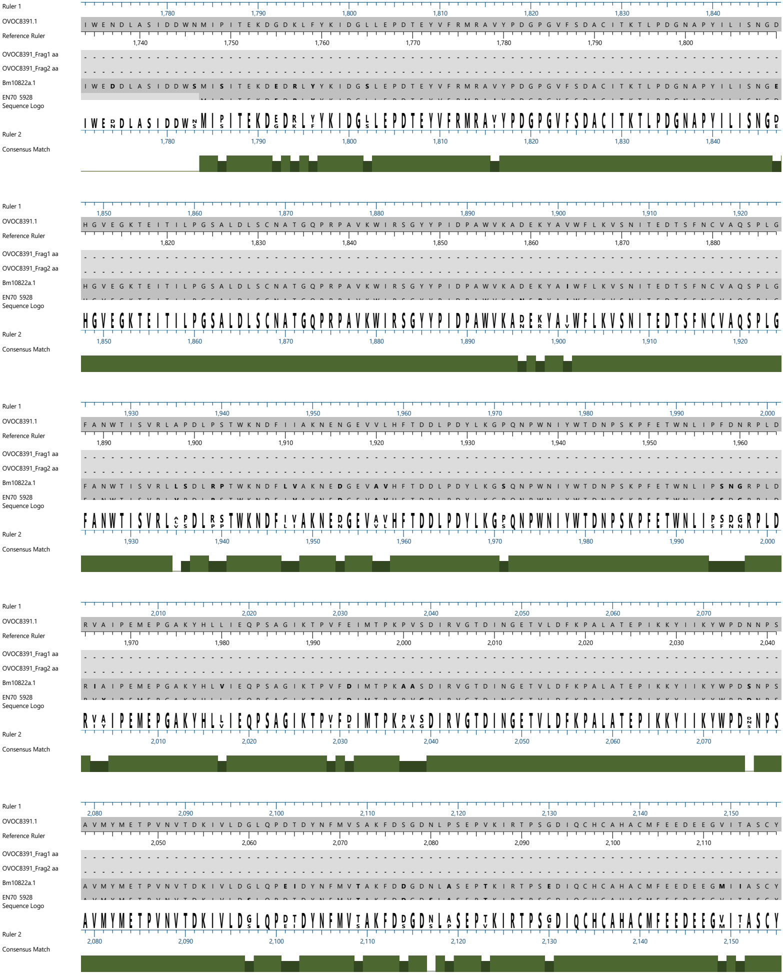

Ruler 1
OVOC8391.1
Reference Ruler
OVOC8391_Frag1 aa
OVOC8391_Frag2 aa
Bm10822a.1
EN70 5928
Sequence Logo
Ruler 2
Consensus Match
1,780 1,790 1,800 1,810 1,820 1,830 1,840
I W E N D L A S I D D W N M I P I T E K D G D K L F Y K I D G L L E P D T E Y V F R M R A V Y P D G P G V F S D A C I T K T L P D G N A P Y I L I S N G D
1,740 1,750 1,760 1,770 1,780 1,790 1,800
I W E D D L A S I D D W S M I S I T E K D E D R L Y Y K I D G S L E P D T E Y V F R M R A V Y P D G P G V F S D A C I T K T L P D G N A P Y I L I S N G E
1,850 1,860 1,870 1,880 1,890 1,900 1,910 1,920
H G V E G K T E I T I L P G S A L D L S C N A T G Q P R P A V K W I R S G Y Y P I D P A W V K A D E K Y A V W F L K V S N I T E D T S F N C V A Q S P L G
1,820 1,830 1,840 1,850 1,860 1,870 1,880
H G V E G K T E I T I L P G S A L D L S C N A T G Q P R P A V K W I R S G Y Y P I D P A W V K A D E K Y A I W F L K V S N I T E D T S F N C V A Q S P L G
1,930 1,940 1,950 1,960 1,970 1,980 1,990 2,000
F A N W T I S V R L A P D L P S T W K N D F I I A K N E N G E V V L H F T D D L P D Y L K G P Q N P W N I Y W T D N P S K P F E T W N L I P F D N R P L D
1,890 1,900 1,910 1,920 1,930 1,940 1,950 1,960
F A N W T I S V R L L S D L R P T W K N D F L V A K N E D G E V A V H F T D D L P D Y L K G S Q N P W N I Y W T D N P S K P F E T W N L I P S N G R P L D
2,010 2,020 2,030 2,040 2,050 2,060 2,070
R V A I P E M E P G A K Y H L L I E Q P S A G I K T P V F E I M T P K P V S D I R V G T D I N G E T V L D F K P A L A T E P I K K Y I I K Y W P D N N P S
1,970 1,980 1,990 2,000 2,010 2,020 2,030 2,040
R I A I P E M E P G A K Y H L V I E Q P S A G I K T P V F D I M T P K A A S D I R V G T D I N G E T V L D F K P A L A T E P I K K Y I I K Y W P D S N P S
2,080 2,090 2,100 2,110 2,120 2,130 2,140 2,150
A V M Y M E T P V N V T D K I V L D G L Q P D T D Y N F M V S A K F D S G D N L P S E P V K I R T P S G D I Q C H C A H A C M F E E D E E G V I T A S C Y
2,050 2,060 2,070 2,080 2,090 2,100 2,110
A V M Y M E T P V N V T D K I V L D G L Q P E I D Y N F M V T A K F D D G D N L A S E P T K I R T P S E D I Q C H C A H A C M F E E D E E G M I I A S C Y

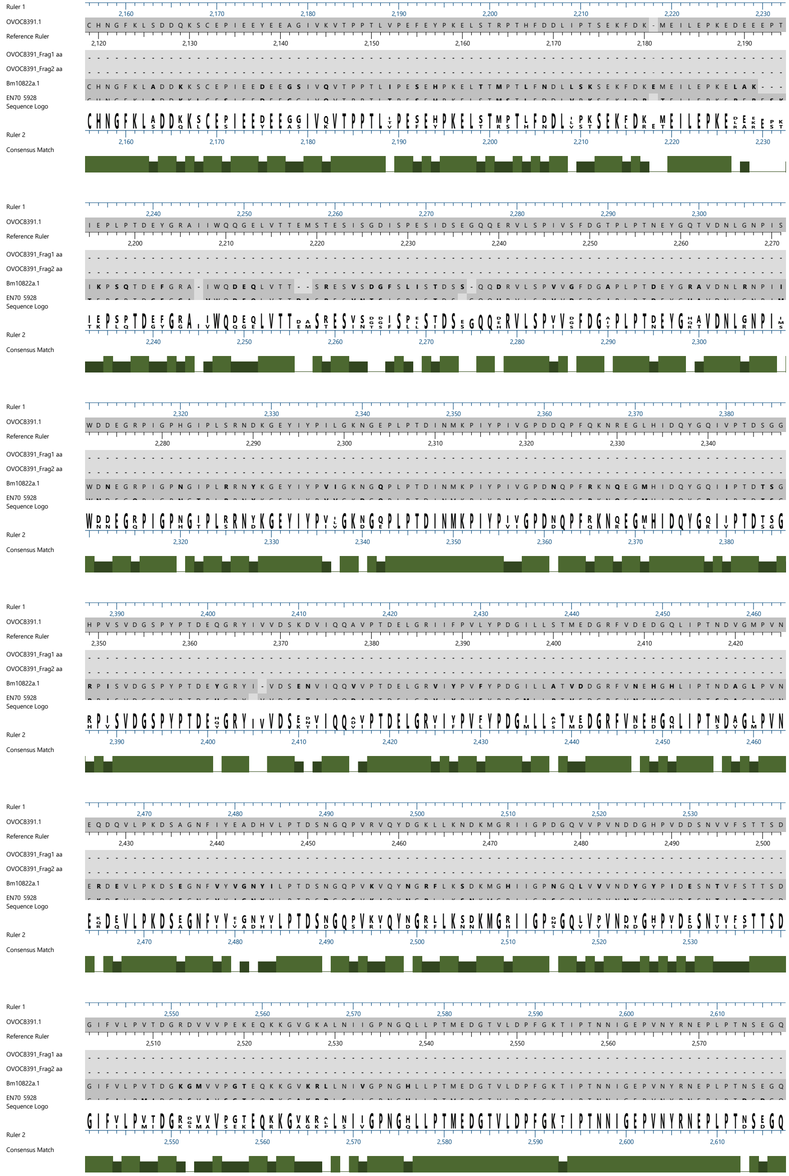
Ruler 1
OVOC8391.1
C H N G F K L S D D Q K S C E P I E E Y E E A G I V K V T P P T L V P E F E Y P K E L S T R P T H F D D L I P T S E K F D K - M E I L E P K E D E E E P T
Reference Ruler
OVOC8391_Frag1 aa
OVOC8391_Frag2 aa
Bm10822a.1
C H N G F K L A D D K K S C E P I E E D E E G S I V Q V T P P T L I P E S E H P K E L T T M P T L F N D L L S K S E K F D K E M E I L E P K E L A K - - -
EN70 5928
Sequence Logo
Ruler 2
Consensus Match
OVOC8391.1
I E P L P T D E Y G R A I I W Q Q G E L V T T E M S T E S I S G D I S P E S I D S E G Q Q E R V L S P I V S F D G T P L P T N E Y G Q T V D N L G N P I S
Bm10822a.1
I K P S Q T D E F G R A - I W Q D E Q L V T T - - S R E S V S D G F S L I S T D S S - Q Q D R V L S P V V G F D G A P L P T D E Y G R A V D N L R N P I I
OVOC8391.1
W D D E G R P I G P H G I P L S R N D K G E Y I Y P I L G K N G E P L P T D I N M K P I Y P I V G P D D Q P F Q K N R E G L H I D Q Y G Q I V P T D S G G
Bm10822a.1
W D N E G R P I G P N G I P L R R N Y K G E Y I Y P V I G K N G Q P L P T D I N M K P I Y P I V G P D N Q P F R K N Q E G M H I D Q Y G Q I I P T D T S G
OVOC8391.1
H P V S V D G S P Y P T D E Q G R Y I V V D S K D V I Q Q A V P T D E L G R I I F P V L Y P D G I L L S T M E D G R F V D E D G Q L I P T N D V G M P V N
Bm10822a.1
R P I S V D G S P Y P T D E Y G R Y I - V D S E N V I Q Q V V P T D E L G R V I Y P V F Y P D G I L L A T V D D G R F V N E H G H L I P T N D A G L P V N
OVOC8391.1
E Q D Q V L P K D S A G N F I Y E A D H V L P T D S N G Q P V R V Q Y D G K L L K N D K M G R I I G P D G Q V V P V N D D G H P V D D S N V V F S T T S D
Bm10822a.1
E R D E V L P K D S E G N F V Y V G N Y I L P T D S N G Q P V K V Q Y N G R F L K S D K M G H I I G P N G Q L V V V N D Y G Y P I D E S N T V F S T T S D
OVOC8391.1
G I F V L P V T D G R D V V V P E K E Q K K G V G K A L N I I G P N G Q L L P T M E D G T V L D P F G K T I P T N N I G E P V N Y R N E P L P T N S E G Q
Bm10822a.1
G I F V L P V T D G K G M V V P G T E Q K K G V K R L L N I V G P N G H L L P T M E D G T V L D P F G K T I P T N N I G E P V N Y R N E P L P T N S E G Q

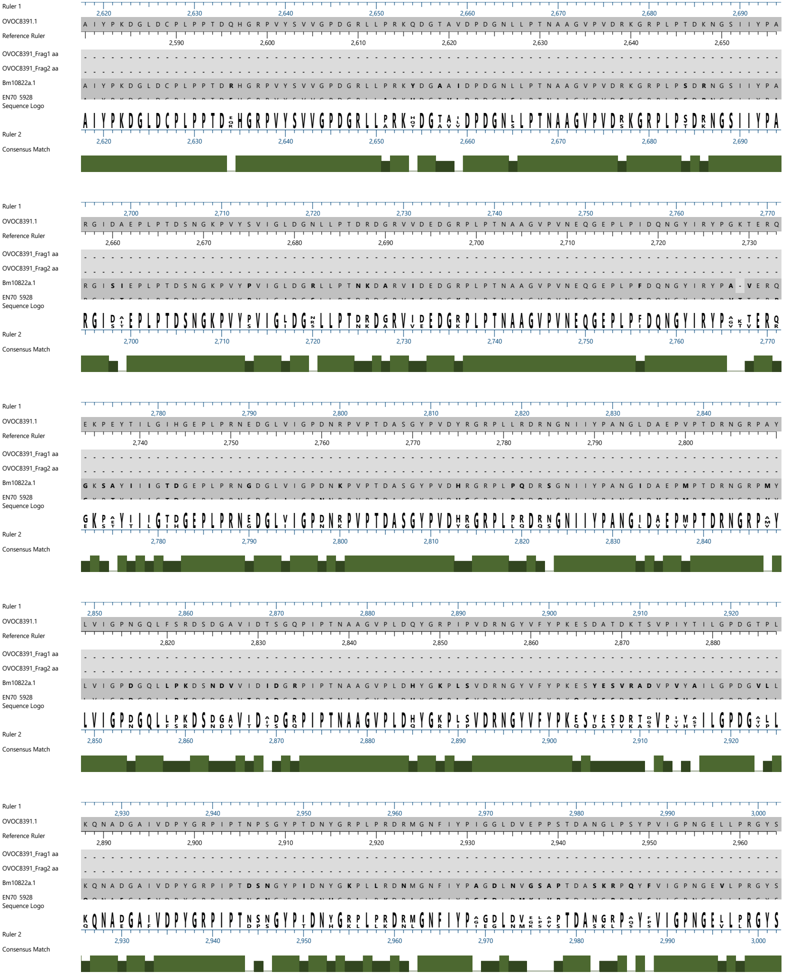
Ruler 1
OVOC8391.1
Reference Ruler
OVOC8391_Frag1 aa
OVOC8391_Frag2 aa
Bm10822a.1
EN70 5928
Sequence Logo
Ruler 2
Consensus Match
A I Y P K D G L D C P L P P T D Q H G R P V Y S V V G P D G R L L P R K Q D G T A V D P D G N L L P T N A A G V P V D R K G R P L P T D K N G S I I Y P A
A I Y P K D G L D C P L P P T D R H G R P V Y S V V G P D G R L L P R K Y D G A A I D P D G N L L P T N A A G V P V D R K G R P L P S D R N G S I I Y P A
R G I D A E P L P T D S N G K P V Y S V I G L D G N L L P T D R D G R V V D E D G R P L P T N A A G V P V N E Q G E P L P I D Q N G Y I R Y P G K T E R Q
R G I S I E P L P T D S N G K P V Y P V I G L D G R L L P T N K D A R V I D E D G R P L P T N A A G V P V N E Q G E P L P F D Q N G Y I R Y P A - V E R Q
E K P E Y T I L G I H G E P L P R N E D G L V I G P D N R P V P T D A S G Y P V D Y R G R P L L R D R N G N I I Y P A N G L D A E P V P T D R N G R P A Y
G K S A Y I I I G T D G E P L P R N G D G L V I G P D N K P V P T D A S G Y P V D H R G R P L P Q D R S G N I I Y P A N G I D A E P M P T D R N G R P M Y
L V I G P N G Q L F S R D S D G A V I D T S G Q P I P T N A A G V P L D Q Y G R P I P V D R N G Y V F Y P K E S D A T D K T S V P I Y T I L G P D G T P L
L V I G P D G Q L L P K D S N D V V I D I D G R P I P T N A A G V P L D H Y G K P L S V D R N G Y V F Y P K E S Y E S V R A D V P V Y A I L G P D G V L L
K Q N A D G A I V D P Y G R P I P T N P S G Y P T D N Y G R P L P R D R M G N F I Y P I G G L D V E P P S T D A N G L P S Y P V I G P N G E L L P R G Y S
K Q N A D G A I V D P Y G R P I P T D S N G Y P I D N Y G K P L L R D N M G N F I Y P A G D L N V G S A P T D A S K R P Q Y F V I G P N G E V L P R G Y S

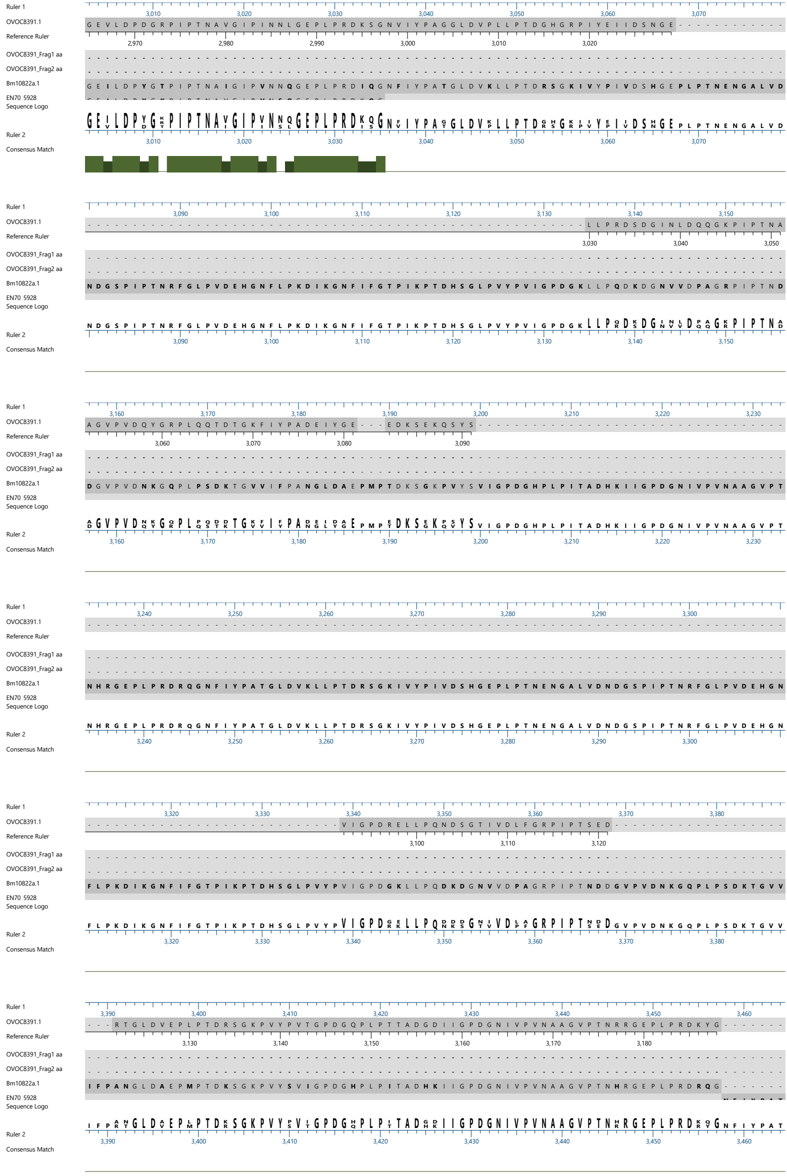
Ruler 1
OVOC8391.1
Reference Ruler
OVOC8391_Frag1 aa
OVOC8391_Frag2 aa
Bm10822a.1
EN70 5928
Sequence Logo
Ruler 2
Consensus Match

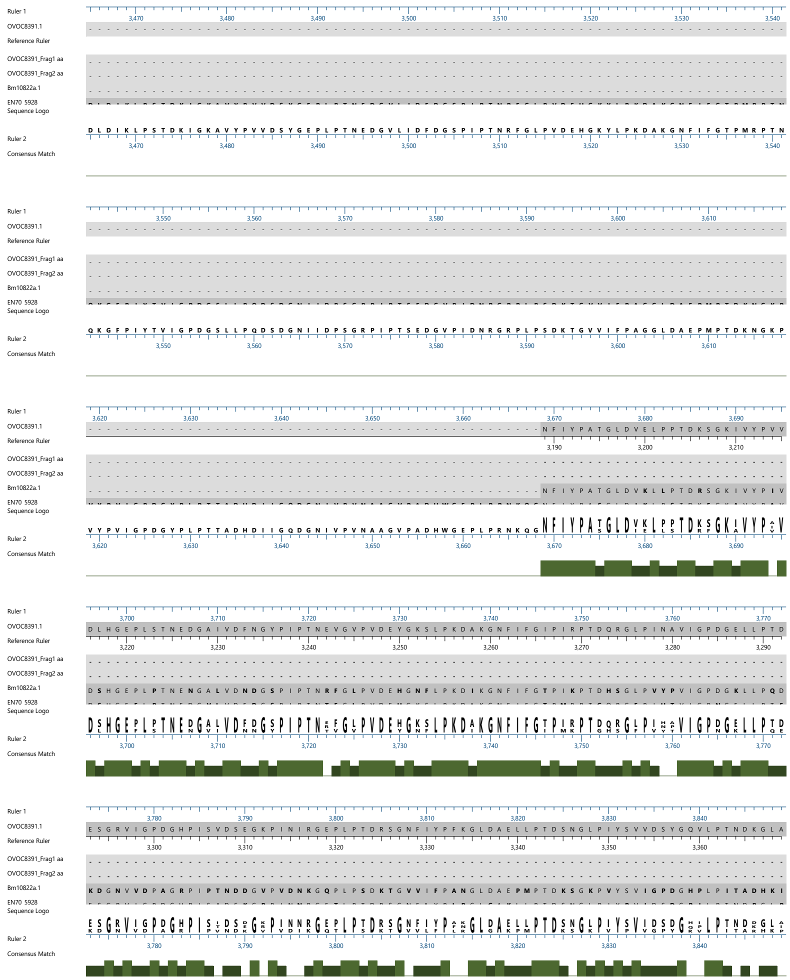

Ruler 1
OVOC8391.1
Reference Ruler
OVOC8391_Frag1 aa
OVOC8391_Frag2 aa
Bm10822a.1
EN70 5928
Sequence Logo
Ruler 2
Consensus Match
3,470 3,480 3,490 3,500 3,510 3,520 3,530 3,540
D L D I K L P S T D K I G K A V Y P V V D S Y G E P L P T N E D G V L I D F D G S P I P T N R F G L P V D E H G K Y L P K D A K G N F I F G T P M R P T N
3,550 3,560 3,570 3,580 3,590 3,600 3,610
Q K G F P I Y T V I G P D G S L L P Q D S D G N I I D P S G R P I P T S E D G V P I D N R G R P L P S D K T G V V I F P A G G L D A E P M P T D K N G K P
3,620 3,630 3,640 3,650 3,660 3,670 3,680 3,690
N F I Y P A T G L D V E L P P T D K S G K I V Y P V V
3,190 3,200 3,210
N F I Y P A T G L D V K L L P T D R S G K I V Y P I V
V Y P V I G P D G Y P L P T T A D H D I I G Q D G N I V P V N A A G V P A D H W G E P L P R N K Q G
3,700 3,710 3,720 3,730 3,740 3,750 3,760 3,770
D L H G E P L S T N E D G A I V D F N G Y P I P T N E V G V P V D E Y G K S L P K D A K G N F I F G I P I R P T D Q R G L P I N A V I G P D G E L L P T D
3,220 3,230 3,240 3,250 3,260 3,270 3,280 3,290
D S H G E P L P T N E N G A L V D N D G S P I P T N R F G L P V D E H G N F L P K D I K G N F I F G T P I K P T D H S G L P V Y P V I G P D G K L L P Q D
3,780 3,790 3,800 3,810 3,820 3,830 3,840
E S G R V I G P D G H P I S V D S E G K P I N I R G E P L P T D R S G N F I Y P F K G L D A E L L P T D S N G L P I Y S V V D S Y G Q V L P T N D K G L A
3,300 3,310 3,320 3,330 3,340 3,350 3,360
K D G N V V D P A G R P I P T N D D G V P V D N K G Q P L P S D K T G V V I F P A N G L D A E P M P T D K S G K P V Y S V I G P D G H P L P I T A D H K I

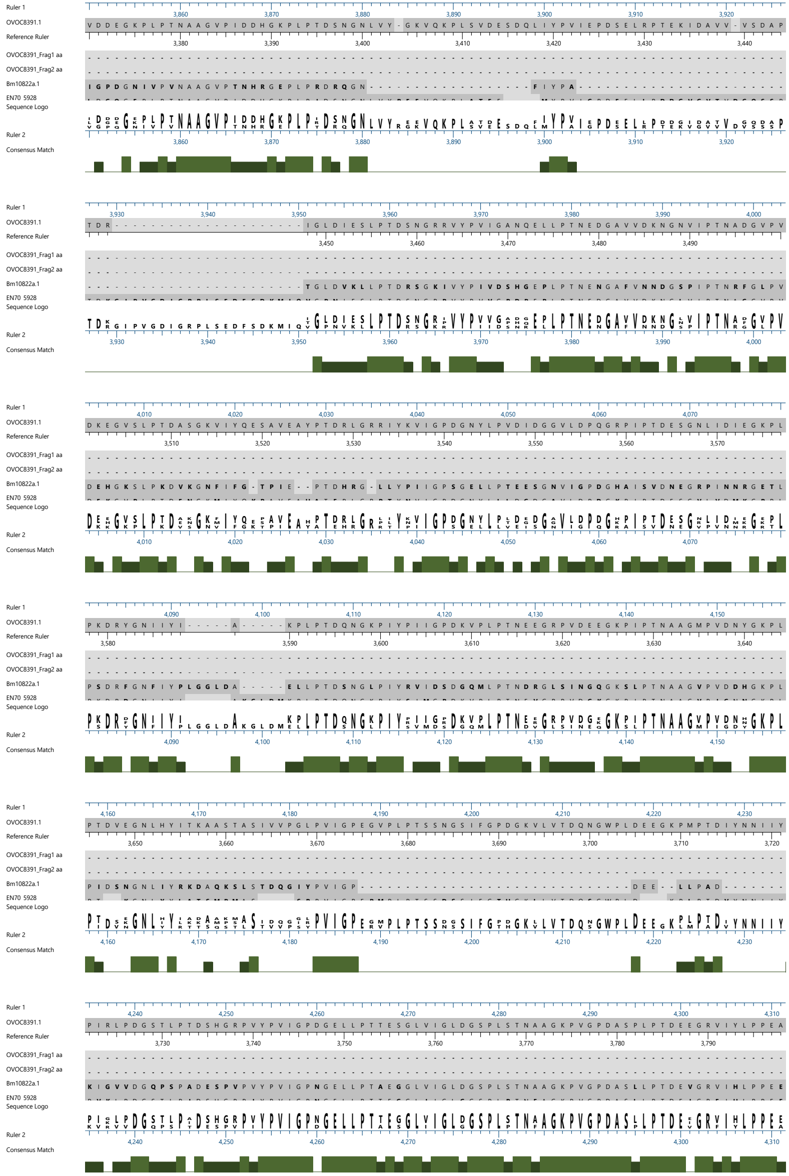
Ruler 1
3,860 3,870 3,880 3,890 3,900 3,910 3,920
OVOC8391.1
VDDEGKPLPTNAAGVPIDDHGKPLPTDSNGNLVY-GKVQKPLSVDESDQLIYPVIEPDSELRPTEKIDAVV-VSDAP
Reference Ruler
3,380 3,390 3,400 3,410 3,420 3,430 3,440
OVOC8391_Frag1 aa
OVOC8391_Frag2 aa
Bm10822a.1
IGPDGNIVPVNAAGVPTNHRGEPLPRDRQGN------------------FIYPA
EN70 5928
Sequence Logo
Ruler 2
Consensus Match
3,930 3,940 3,950 3,960 3,970 3,980 3,990 4,000
TDR----------------------IGLDIESLPTDSNGRRVYPVIGANQELLPTNEDGAVVDKNGNVIPTNADGVPV
3,450 3,460 3,470 3,480 3,490
-------------------------TGLDVKLLPTDRSGKIVYPIVDSHGEPLPTNENGAFVNNDGSPIPTNRFGLPV
4,010 4,020 4,030 4,040 4,050 4,060 4,070
DKEGVSLPTDASGKVIYQESAVEAYPTDRLGRRIYKVIGPDGNYLPVDIDGGVLDPQGRPIPTDESGNLIDIEGKPL
3,510 3,520 3,530 3,540 3,550 3,560 3,570
DEHGKSLPKDVKGNFIFG-TPIE--PTDHRG-LLYPIIGPSGELLPTEESGNVIGPDGHAISVDNEGRPINNRGETL
4,090 4,100 4,110 4,120 4,130 4,140 4,150
PKDRYGNIIYI-----A-----KPLPTDQNGKPIYPIIGPDKVPLPTNEEGRPVDEEGKPIPTNAAGMPVDNYGKPL
3,580 3,590 3,600 3,610 3,620 3,630 3,640
PSDRFGNFIYPLGGLDA-----ELLPTDSNGLPIYRVIDSDGQMLPTNDRGLSINGQGKSLPTNAAGVPVDDHGKPL
4,160 4,170 4,180 4,190 4,200 4,210 4,220 4,230
PTDVEGNLHYITKAASTASIVVPGLPVIGPEGVPLPTSSNGSIFGPDGKVLVTDQNGWPLDEEGKPMPTDIYNNIIY
3,650 3,660 3,670 3,680 3,690 3,700 3,710 3,720
PIDSNGNLIYRKDAQKSLSTDQGIYPVIGP------------------------DEE--LLPAD-----
4,240 4,250 4,260 4,270 4,280 4,290 4,300 4,310
PIRLPDGSTLPTDSHGRPVYPVIGPDGELLPTTESGLVIGLDGSPLSTNAAGKPVGPDASPLPTDEEGRVIYLPPEA
3,730 3,740 3,750 3,760 3,770 3,780 3,790
KIGVVDGQPSPADESPVPVYPVIGPNGELLPTAEGGLVIGLDGSPLSTNAAGKPVGPDASLLPTDEVGRVIHLPPEE

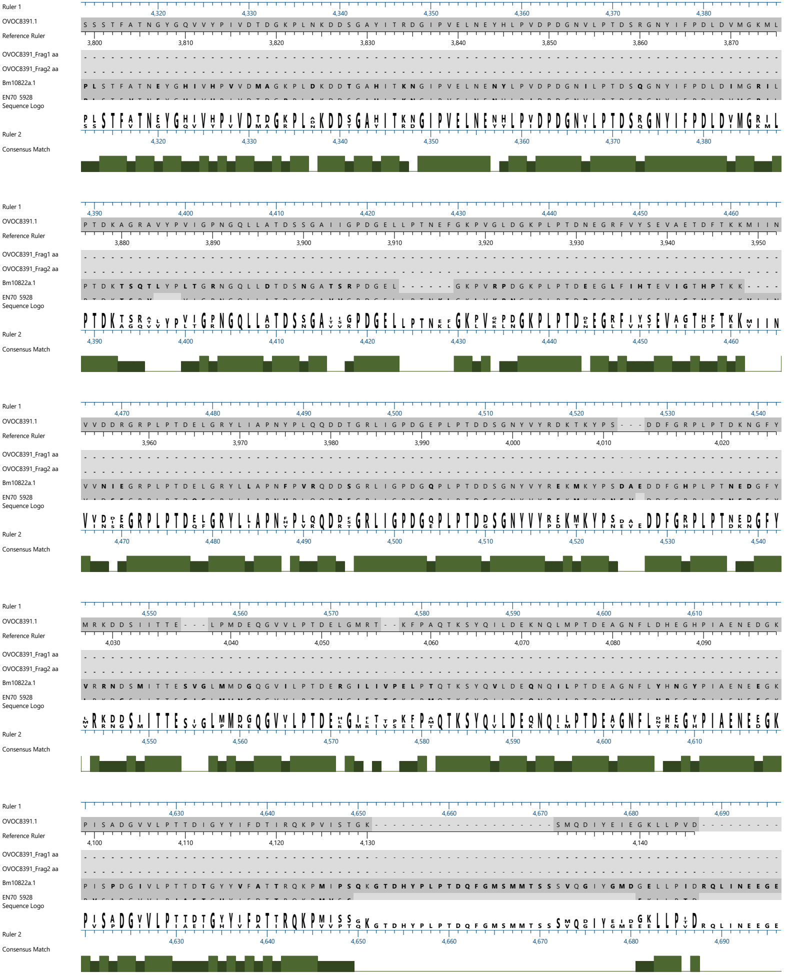
Ruler 1
OVOC8391.1
Reference Ruler
OVOC8391_Frag1 aa
OVOC8391_Frag2 aa
Bm10822a.1
EN70 5928
Sequence Logo
Ruler 2
Consensus Match

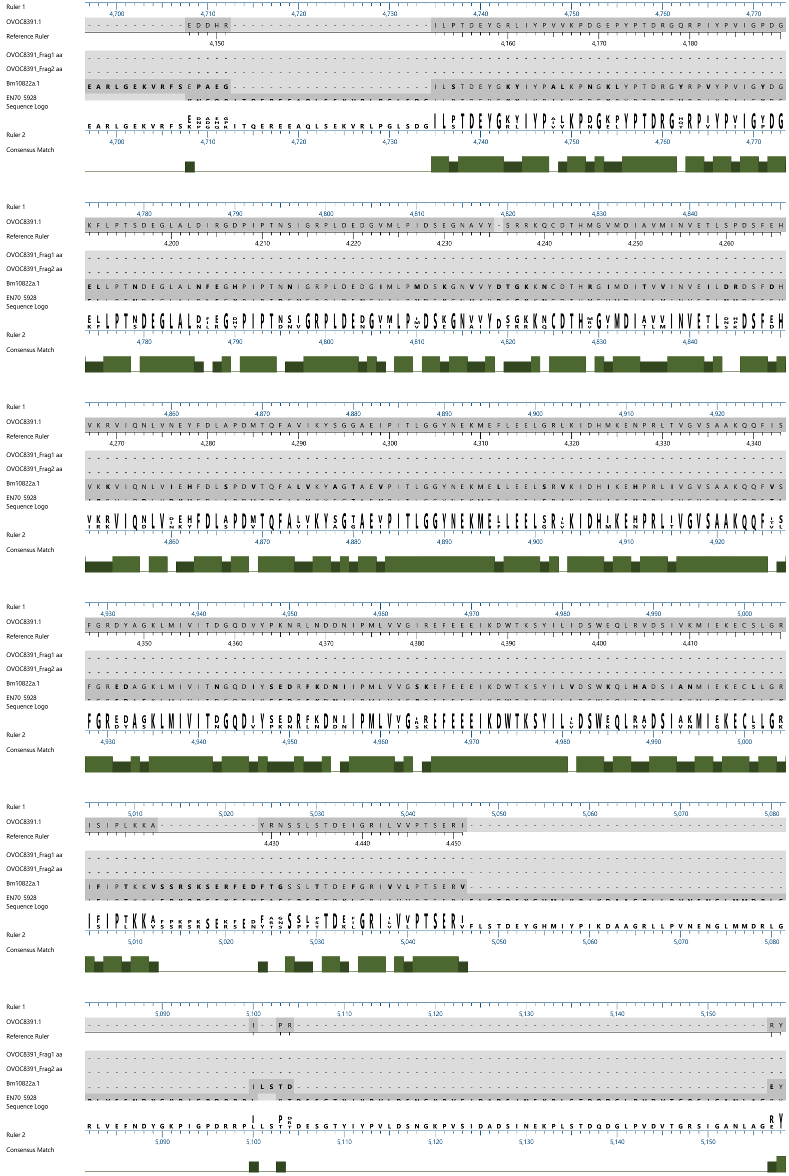
Ruler 1
OVOC8391.1
Reference Ruler
OVOC8391_Frag1 aa
OVOC8391_Frag2 aa
Bm10822a.1
EN70 5928
Sequence Logo
Ruler 2
Consensus Match

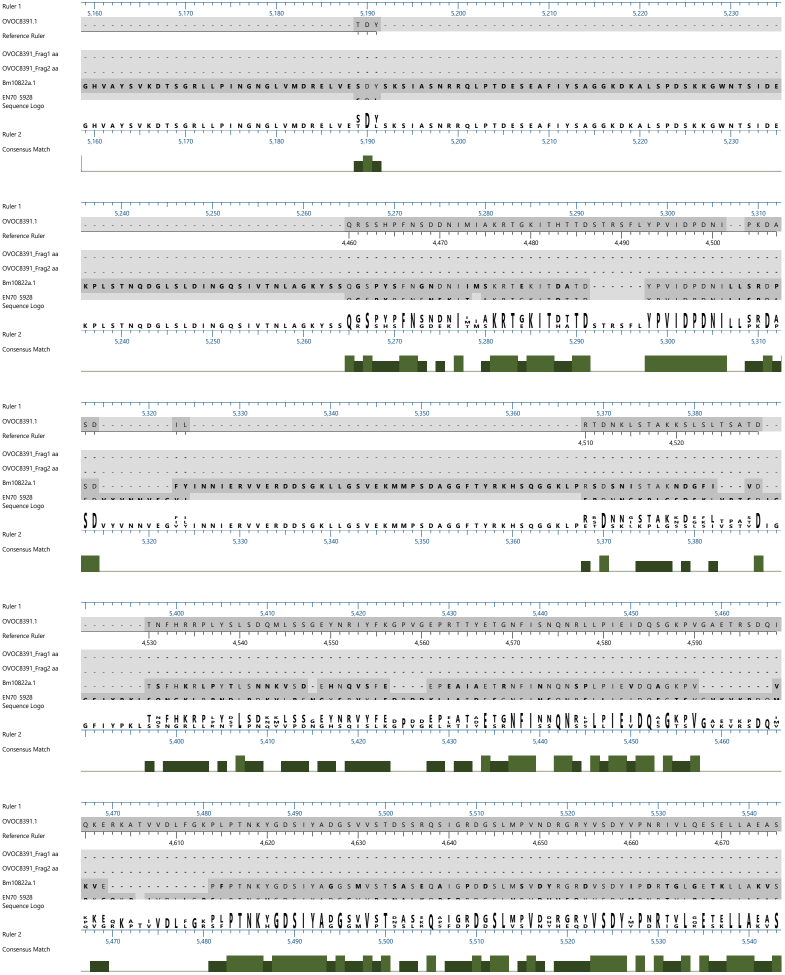
Ruler 1
OVOC8391.1
Reference Ruler
OVOC8391_Frag1 aa
OVOC8391_Frag2 aa
Bm10822a.1
EN70 5928
Sequence Logo
Ruler 2
Consensus Match

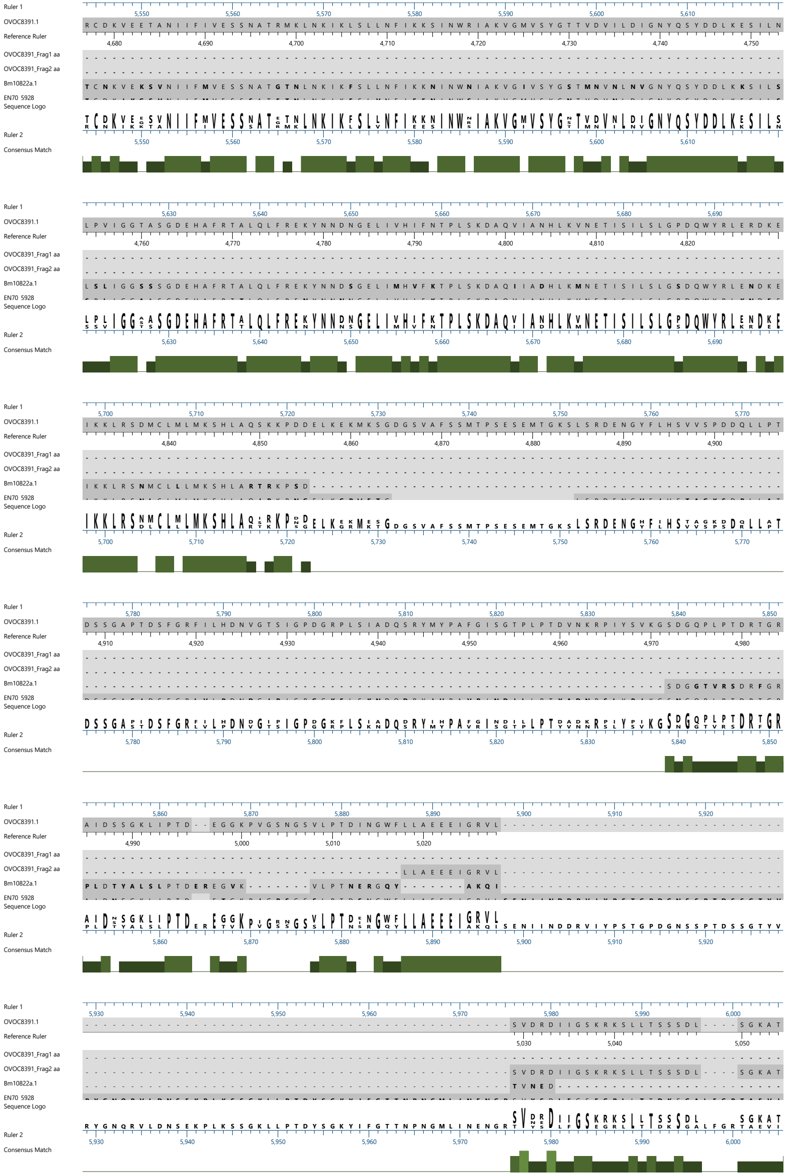
Ruler 1
OVOC8391.1
Reference Ruler
OVOC8391_Frag1 aa
OVOC8391_Frag2 aa
Bm10822a.1
EN70 5928
Sequence Logo
Ruler 2
Consensus Match
5,550 5,560 5,570 5,580 5,590 5,600 5,610
R C D K V E E T A N I I F I V E S S N A T R M K L N K I K L S L L N F I K K S I N W R I A K V G M V S Y G T T V D V I L D I G N Y Q S Y D D L K E S I L N
4,680 4,690 4,700 4,710 4,720 4,730 4,740 4,750
T C N K V E K S V N I I F M V E S S N A T G T N L N K I K F S L L N F I K K N I N W N I A K V G I V S Y G S T M N V N L N V G N Y Q S Y D D L K K S I L S
5,630 5,640 5,650 5,660 5,670 5,680 5,690
L P V I G G T A S G D E H A F R T A L Q L F R E K Y N N D N G E L I V H I F N T P L S K D A Q V I A N H L K V N E T I S I L S L G P D Q W Y R L E R D K E
4,760 4,770 4,780 4,790 4,800 4,810 4,820
L S L I G G S S S G D E H A F R T A L Q L F R E K Y N N D S G E L I M H V F K T P L S K D A Q I I A D H L K M N E T I S I L S L G S D Q W Y R L E N D K E
5,700 5,710 5,720 5,730 5,740 5,750 5,760 5,770
I K K L R S D M C L M L M K S H L A Q S K K P D D E L K E K M K S G D G S V A F S S M T P S E S E M T G K S L S R D E N G Y F L H S V V S P D D Q L L P T
4,840 4,850 4,860 4,870 4,880 4,890 4,900
I K K L R S N M C L L L M K S H L A R T R K P S D
5,780 5,790 5,800 5,810 5,820 5,830 5,840 5,850
D S S G A P T D S F G R F I L H D N V G T S I G P D G R P L S I A D Q S R Y M Y P A F G I S G T P L P T D V N K R P I Y S V K G S D G Q P L P T D R T G R
4,910 4,920 4,930 4,940 4,950 4,960 4,970 4,980
S D G G T V R S D R F G R
5,860 5,870 5,880 5,890 5,900 5,910 5,920
A I D S S G K L I P T D E G G K P V G S N G S V L P T D I N G W F L L A E E E I G R V L
4,990 5,000 5,010 5,020
L L A E E E I G R V L
P L D T Y A L S L P T D E R E G V K V L P T N E R G Q Y A K Q I
5,930 5,940 5,950 5,960 5,970 5,980 5,990 6,000
S V D R D I I G S K R K S L L T S S S D L S G K A T
5,030 5,040 5,050
S V D R D I I G S K R K S L L T S S S D L S G K A T
T V N E D

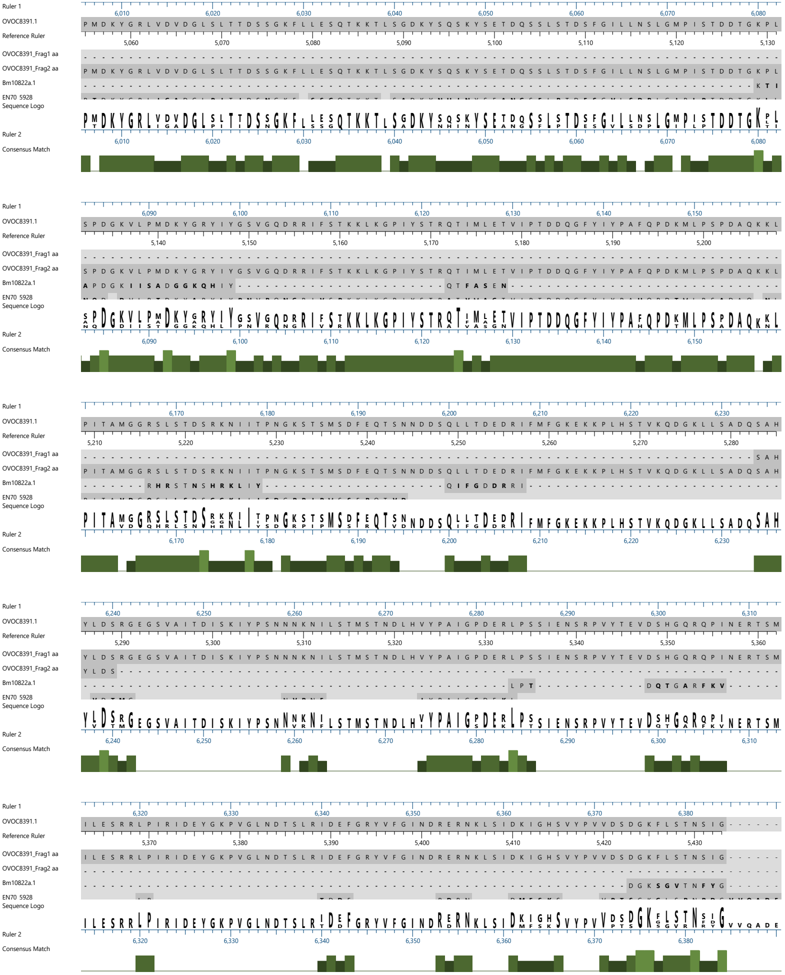
Ruler 1
OVOC8391.1
Reference Ruler
OVOC8391_Frag1 aa
OVOC8391_Frag2 aa
Bm10822a.1
EN70 5928
Sequence Logo
Ruler 2
Consensus Match
P M D K Y G R L V D V D G L S L T T D S S G K F L L E S Q T K K T L S G D K Y S Q S K Y S E T D Q S S L S T D S F G I L L N S L G M P I S T D D T G K P L
S P D G K V L P M D K Y G R Y I Y G S V G Q D R R I F S T K K L K G P I Y S T R Q T I M L E T V I P T D D Q G F Y I Y P A F Q P D K M L P S P D A Q K K L
P I T A M G G R S L S T D S R K N I I T P N G K S T S M S D F E Q T S N N D D S Q L L T D E D R I F M F G K E K K P L H S T V K Q D G K L L S A D Q S A H
Y L D S R G E G S V A I T D I S K I Y P S N N N K N I L S T M S T N D L H V Y P A I G P D E R L P S S I E N S R P V Y T E V D S H G Q R Q P I N E R T S M
I L E S R R L P I R I D E Y G K P V G L N D T S L R I D E F G R Y V F G I N D R E R N K L S I D K I G H S V Y P V V D S D G K F L S T N S I G

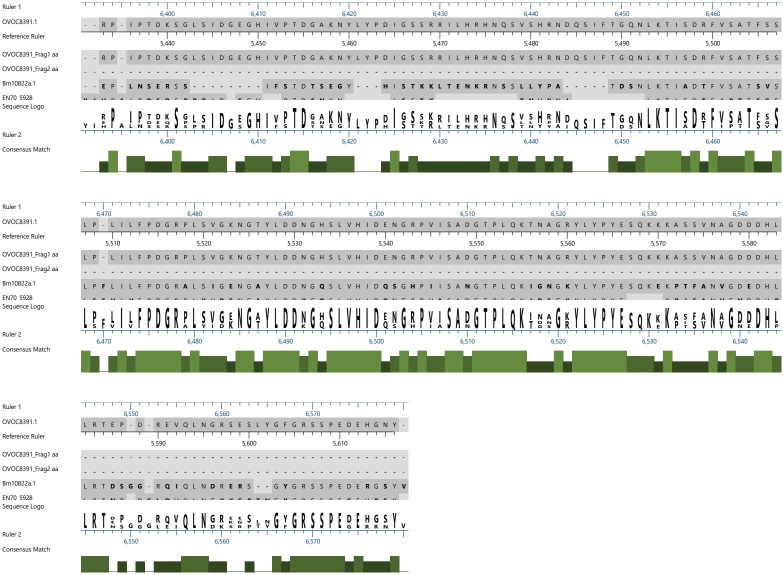

Ruler 1
OVOC8391.1
- - R P - I P T D K S G L S I D G E G H I V P T D G A K N Y L Y P D I G S S R R I L H R H N Q S V S H R N D Q S I F T G Q N L K T I S D R F V S A T F S S
Reference Ruler
OVOC8391_Frag1 aa
OVOC8391_Frag2 aa
Bm10822a.1
- - E P - L N S E R S S - - - - - - - - I F S T D T S E G Y - - - H I S T K K L T E N K R N S S L L Y P A - - - - - T D S N L K T I A D T F V S A T S V S
EN70 5928
Sequence Logo
Ruler 2
Consensus Match
Ruler 1
OVOC8391.1
L P - L I L F P D G R P L S V G K N G T Y L D D N G H S L V H I D E N G R P V I S A D G T P L Q K T N A G R Y L Y P Y E S Q K K K A S S V N A G D D D H L
Reference Ruler
OVOC8391_Frag1 aa
OVOC8391_Frag2 aa
Bm10822a.1
L P F L I L F P D G R A L S I G E N G A Y L D D N G Q S L V H I D Q S G H P I I S A N G T P L Q K I G N G K Y L Y P Y E S Q K E K P T F A N V G D E D H L
EN70 5928
Sequence Logo
Ruler 2
Consensus Match
Ruler 1
OVOC8391.1
L R T E P - D - R E V Q L N G R S E S L Y G F G R S S P E D E H G N Y -
Reference Ruler
OVOC8391_Frag1 aa
OVOC8391_Frag2 aa
Bm10822a.1
L R T D S G G - R Q I Q L N D R E R S - - G Y G R S S P E D E R G S Y V
EN70 5928
Sequence Logo
Ruler 2
Consensus Match
